# Brain Tumors and Electroconvulsive Therapy: A Literature Overview of the Last 80 Years

**DOI:** 10.3389/fneur.2020.00723

**Published:** 2020-07-31

**Authors:** Jozef Buday, Jakub Albrecht, Tadeas Mareš, Gabriela Podgorná, Klara Horáčková, Lucie Kališová, Jiri Raboch, Martin Anders

**Affiliations:** Department of Psychiatry, First Faculty of Medicine Charles University and General University Hospital, Prague, Czechia

**Keywords:** ECT, brain tumor, abnormal recovery, ECT safety, ECT and cancer

## Abstract

The safety and efficacy of electroconvulsive therapy (ECT) in patients with a brain tumor have been debated in the past without a clear conclusion. In the last large review published by Maltbie et al. in 1980, it was concluded that the presence of an intracranial mass should be considered an absolute contraindication to ECT. In our updated review, we investigated a total of 33 published and indexed case reports, case report series, and reviews of 75 individual patients who underwent ECT in the presence of a brain tumor over the last 80 years. Mounting case reports after the original Maltbie et al. review show that it is feasible to apply this method safely in patients with benign or otherwise clinically insignificant lesions. Certain precautionary measures, such as dexamethasone or phenytoin application before ECT, could lead to a further minimalization or even absence of adverse effects, particularly in higher risk individuals.

## Introduction

The safety and efficacy of electroconvulsive therapy (ECT) in patients with a brain tumor were debated in the past without a clear conclusion. In the last large review published by Maltbie et al. (1) in 1980, it was deemed that the presence of an intracranial mass should be considered an absolute contraindication to ECT. In our updated review, we investigated a total of 33 published and indexed case reports, case report series, and reviews of 75 individual patients who underwent ECT in the presence of a brain tumor over the last 80 years.

## Study Aim

The main aim of our review was to find out whether the original conclusion of Maltbie et al. (1) (the presence of a brain tumor should be considered an absolute contraindication to ECT) can be challenged by collecting and analyzing all case reports, case report series, or mini reviews that emerged on this topic since the publication of this review 40 years ago. Our secondary goal was to provide a comprehensive overview about the occurrence and severity of adverse effects (AEs) after ECT in patients with a brain tumor and find possible methods to increase the safety of this important treatment modality in current scientific literature.

## Methods

**Figure 1:**
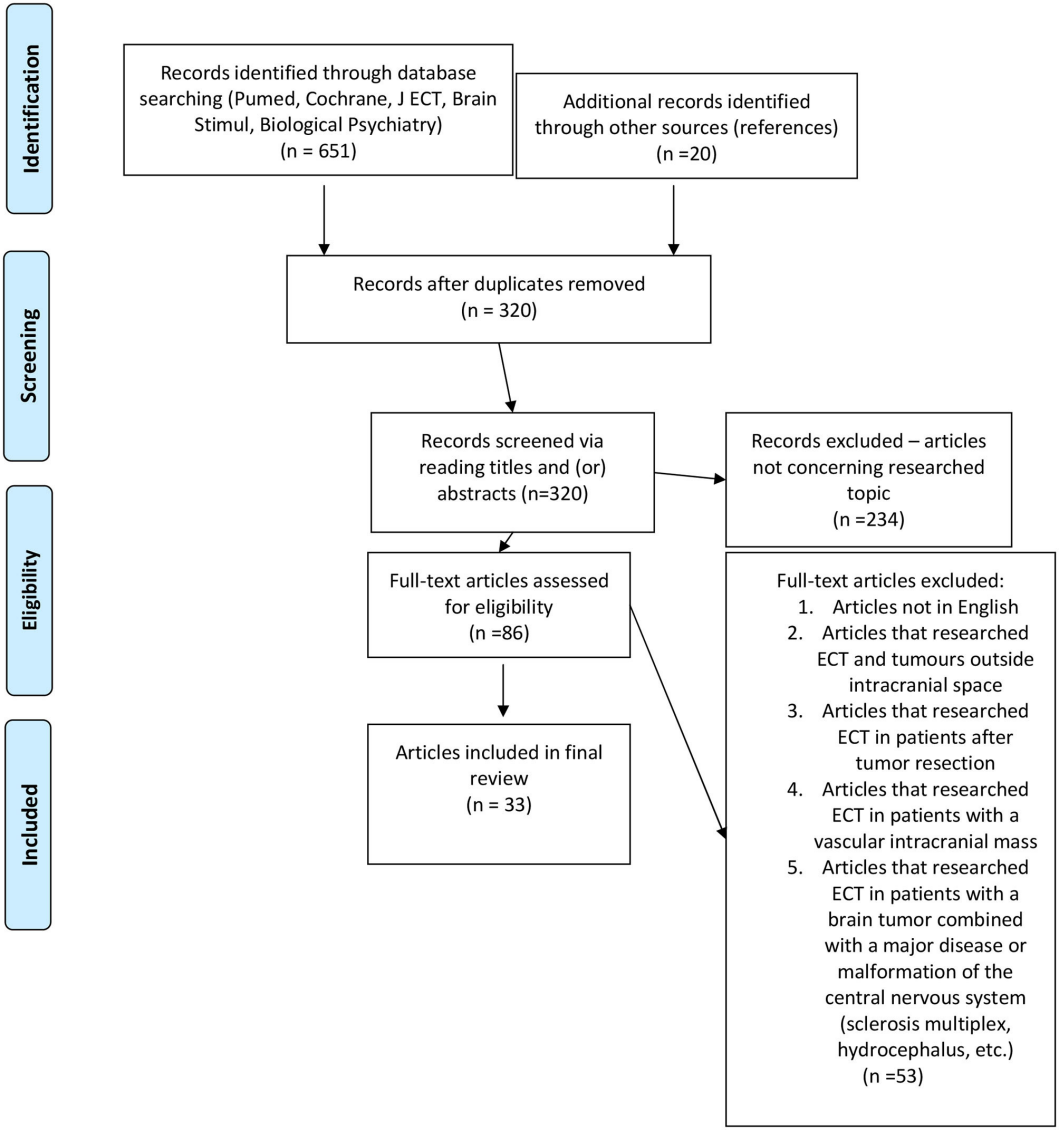
PRISMA flow chart of our search strategy. (“File:PRISMA flow chart for Wiki Journal of Medicine article.jpg” by Rwatson, 1955 is licensed under CC BY-SA 4.0/the template was used to draw a flow chart on the topic of our article).

We followed the PRISMA statement (2) as a guide for conducting our systematic review. We have searched the electronic databases of Cochrane, PubMed, Journal of ECT, Biological Psychiatry, and Brain Stimulation using combinations of terms “electroconvulsive therapy,” “brain tumor,” and “electroconvulsive therapy,” “intracranial mass,” and “ECT,” “brain tumor.” The search was last updated in May 2020. The electronic search returned 651 abstracts and titles. We screened these abstracts and excluded studies that were duplicates or not directly relevant to the topic of this review. Our inclusion criteria were all original studies—case reports, letters to the Editor, case report series, mini reviews, reviews, or book chapters that reported on patients who underwent ECT with a previously known presence of a brain tumor OR patients in which the brain tumor was discovered during/shortly after a course of ECT. We excluded papers that revolved around the application of ECT in patients after tumor resection, aneurysms, or other vascular malformations; patients with a severe disease or malformation of the central nervous system (hydrocephalus, encephalitis, etc.) (3); and patients with tumors that were outside the intracranial space. Only papers in English were considered for this review. Further details are provided in Figure 1.

The case reports up to 1980 were varied in the details provided—most of them lack the exact description of the type of ECT, stimulation parameters, device, and electrode placement (Table 1) and were already discussed in the Maltbie et al. (1) review. Nonetheless, we have summarized the available data from these individual case reports in Table 1. Because of the large variability in the quality of these articles up to 1980, we have focused our attention to summarize newer case reports, case report series, or mini reviews published between 1984 and 2020 (Table 2).

**Table 1:** ECT in patients with a brain tumor 1946–1980.

**Study**	**Sex**	**Age**	**Neurological symptoms prior to ECT**	**Type of tumor**	**Localization**	**Was tumor presence known before ECT?**	**Type of ECT**	**Number of ECT applications**	**Adverse Effects after ECT**
Rond (4)	M	42	No	Glioma	Pons	No	Unkown	3	Long-term confusion and ataxia
Dressler et al. (5)	F	66	No	Metastasic carcinoma	Left parietal and left occipital lobe	Yes	Unkown	7	No
Shapiro and Goldberg (6)	M	60	No	Meningioma	Frontal lobe	No	Unidirectional current (Reiter machine)	3	Long-term confusion
Shapiro and Goldberg (6)	M	39	Neck and facial pain	Meningioma	Right sphenoidal ridge	No	Unidirectional current (Reiter machine)	6	Long-term confusion
Shapiro and Goldberg (6)	M	70	Dyscalculia, hand extiction	Glioblastoma multiforme	Right fronto-temporal region	No	Unidirectional current (Reiter machine)	7	Long-term confusion, aphasia
Shapiro and Goldberg (6)	M	69	Memory defects, truncal ataxia	Glioblastoma	Corpus callosum	No	Unidirectional current (Reiter machine)	5	Long-term confusion
Shapiro and Goldberg (6)	M	47	Paresis of the right lower extremity, severe recurrent headaches	Glioblastoma	Left frontal lobe	No	unidirectional current (Reiter machine)	4	Aphasia
Shapiro and Goldberg (6)	M	56	No	Glioblastoma	Left temporo-parietal region	No	Unidirectional current (Reiter machine)	2	No
Gassel (7)	F	42	Headaches with vomiting	Meningioma	Right frontal lobe	No	Unknown	1	Long-term confusion, weakness in all limbs
Gassel (7)	F	40	Weakness in right lower extremity	Meningioma	Left parietal lobe	No	Unknown	4	Memory deficit complaints, weakness in all limbs
Gassel (7)	F	55	Headaches with vomiting, indeterminate right plantar response	Meningioma	Right parieto-occipital lobe	No	Unknown	4	Aphasia, right miosis, bilateral acute papiledema, spasticity of lower extremities
Gralnick (8)	M	60	No	Meningeal fibroblastoma	Both frontal lobes	No	Unidirectional current	2	Coma
Cole (9)	M	33	No	Oligodendroglioma	Left frontal lobe + both lateral ventricles	No	Unknown	5	Long-term confusion, coma
Delay et al. (10)	F	42	No	Glioma	Left-parieto-temporal region	No	Unknown	6	Secondary seizure after ECT with right-sided lateralisation
Malamud (11)	M	47	Epileptiformn attacks	Astrocytoma	Left temporal lobe	No	Unknown	Unknown	No
Malamud (11)	F	27	No	Astrocytoma	Right fronto-parietal region	No	Unknown	Unknown	Left-side spasticity
Malamud (11)	M	26	No	Glioma	Hippocampus	No	Unknown	Unknown	Epileptic seizures after combined ECT/inzuline treatment
Malamud (11)	F	47	Epilepsy	Astrocytoma	Left temporal lobe	No	Unknown	Unknown	Syncopes after ECT and one record of transient aphasia
Malamud (11)	M	33	Epilepsy	Astrocytoma	Left frontal lobe	No	Unknown	Unknown	Coma
Malamud (11)	F	48	No	Craniopharyngioma	Third ventricle	No	Unknown	Unknown	Dysarthria and subbsequent coma
Malamud (11)	M	28	No	Colloid cyst	Third ventricle	No	Unknown	Unknown	Coma
Paulson (12)	F	49	Headache, dyscomfort in right shoulder	Metastatic carcinoma	Right parietal lobe	No	Unknown	1	Coma
Paulson (12)	M	20	Head and neck pain	Sarcoma	Cerebellum	No	Unknown	6	Coma, bilateral papiloeadema with hemorrhages
Paulson (12)	F	57	Headache, difficulty writing	Metastatic carcinoma	Left frontal lobe	No	Unknown	1	Aphasia, weakness in right upper and lower limb
Kalinowski and Kippins (13)	Unknown	Unknown	Unknown	Unknown	Unknown	No	Unknown	Unknown	Unknown
Maltbie et al. (1)	F	48	Ataxia	Meningioma	Olfactory groove	No	Unknown	1	Coma
Maltbie et al. (1)	M	46	No	Glioblastoma multiforme	Left parietal lobe	No	Unknown	1	Long-term confusion, aphasia, left-sided hyperreflexion
Maltbie et al. (1)	F	48	Epilepsy	Glial tumor	Both frontal lobes	No	Unknown	6	Ataxia, right hemiparesis, withdrawal
Maltbie et al. (1)	M	63	No	Glioblastoma	Occipital region	No	Unknown	12	Persistent headache for 5 months
Maltbie et al. (1)	M	38	No	Oligodendroglioma	Right fronto-temporal region	No	Unknown	15	Sleepiness, left hemiplegia, deviation of the eyes to the left
Maltbie et al. (1)	F	28	No	Dermoid cyst	Cerebello-pontine region	No	Unknown	1	No
Maltbie et al. (1)	F	80	No	Glioblastoma	Left frontal lobe	No	Unknown	1	Right hemiplegia
Waggoner and Bagchi (15)	F	50	No	Menignioma	Left cerebellar hemishpere	No	Unknown	12	No
Waggoner and Bagchi (15)	F	54	No	Glioblastoma multiforme	Right temporo-parietal region	Yes (suspected)	Unknown	3	Left facial paresis, confusion
Waggoner and Bagchi (15)	F	37	Chronic headache and dizziness	Oligodendroglioma	Left frontal lobe	No	Unknown	Unknown	No

**Table 2:** ECT in patients with a brain tumor 1984–2020.

**Study**	**Sex**	**Age**	**Indication (symptoms)**	**Neurological symptoms prior to ECT**	**Type of tumor**	**Size (cm)**	**Localization**	**Was tumor known before ECT**	**Type of ECT**	**no. of ECT**	**Adverse Effects after ECT?**	**Was ECT beneficial?**	**Was ECT discontinued?**	**Notes**
Hsiao and Evans (14)	F	50	Severe depression with psychosis	No	Meningioma	Unknown	Left parietal lobe	Yes	Brief	18	No	Yes	No	
Fried and Mann (16)	M	85	Severe depression with psychosis	No	Meningioma	2.0	Left frontal lobe	Yes	Brief	11	No	Yes	No	Patient received phenytoin prior ECT to prevent prolonged seizure
Greenberg et al. (17)	F	75	Severe depression with psychosis	No	Meningioma	3.0 × 3.0	Fronto-temporo-parietal region	Yes	Brief	8	No	Yes	No	
Goldstein and Richardson (18)	F	74	Severe depression	No	Meningioma	1.0 × 1.5	right frontal lobe	Yes	Brief	6	No	Yes	No	
Malek-Ahmadi and Sedler (19)	F	73	Severe depression	No	Meningioma	0.5	Fronto-parietal region	Yes	Brief	7	No	Yes	No	
Zwil et al. (20)	F	73	Severe depression with psychosis	No	Meningioma	2.0	Cerebello-pontine region	Yes	Brief	10	Post-ictal confusion	Yes	No	Patient received dexamethasone prior ECT
Zwil et al. (20)	F	71	Severe depression with psychosis	No	Meningioma	1.0 × 1.0	Left frontal lobe	Yes	Brief	12	No	Yes	No	
Zwil et al. (20)	F	78	Psychosis	No	Meningioma	2.0	Left parietal lobe	Yes	Brief	10	Post-ictal confusion	Yes	No	
Mattingly et al. (21)	F	75	Severe depression	No	metastatic carcinoma	1.2 × 1.2	cerebellar region	Yes	Brief	11	No	Yes	No	Patient received dexamethasone prior ECT
Holroyd (22)	F	83	catatonia	No	Meningioma	1.5 × 1.5	Left frontal lobe	Yes	Brief	18	No	Yes	No	
Holroyd (22)	F	83	Severe depression	No	Meningioma	2.0 × 2.0	Cerebellar region	Yes	Brief	20	No	Yes	No	
Holroyd (22)	M	76	Severe depression with psychosis	No	Unknown	Unknown	Pitiutary gland	No	Brief	7	No	Yes	Yes	Discontinued after dg. of tumor due to previous reports
Holroyd (22)	F	65	Severe depression	Left hyperreflexia and + Babinski, oral dyskinesia	Meningioma	1.5 × 1.5	Left frontal lobe	Yes	Brief	5	No	Yes	No	
Holroyd (22)	M	60	Psychosis	Left hypoacusia	Acoustic neuroma	4.0	Left posterior fossa	No	Brief	11	No	Yes	No	
Kellner and Rames (23)	F	75	Severe depression with psychosis	No	Meningioma	1.7 × 1.0	Left fronto-temporal region	Yes	Brief	9	No	Yes	No	Patient received dexamethasone prior ECT
Rasmussen et al. (35)	M	71	Psychosis	No	Unknown	1.5 × 1.0	Left parietal lobe	No	Brief	Unknown	No	Yes	No	
Rasmussen et al. (24)	M	59	Severe depression	No	Unknown	0.2	Thalamus	Yes	Brief	9	No	Yes	No	
Rasmussen et al. (24)	M	57	Severe depression	No	Unknown	1.2 parasagitally	Right basal forebrain	No	Brief	6	No	Yes	No	
Rasmussen et al. (24)	F	73	Severe depression	6th nerve palsy	Meningioma	1.0 × 1.7	Ponto-cerebellar region	No	Brief	Unknown	No	Yes	No	
Rasmussen KG et al. 2007	F	80	Severe depression	hypersomnia	cystic mass	3.3 × 2.9 × 3.4	Frontal lobe	No	Brief	Unknown	No	No	Yes	
Rasmussen et al. (24)	M	44	Severe depression	No	Unknown	Unknown	Ponto-medullary region	Yes	Brief	8	No	Yes	No	
Rasmussen et al. (24)	F	33	Severe depression	No	Microadenoma	0.35	Sellar region	Yes	Brief	10	No	Yes	No	
Buday et al. (25)	F	42	Severe depression with psychosis	No	Unknown-likely Meningioma	1.6 × 1.0	Left parietal lobe	No	Ultrabrief	7	Secondary seizure after ECT	Yes	Yes	
Gani and Parvez (26)	F	70	psychosis	No	Meningioma	Unknown	Left frontal lobe	Yes	Ultrabrief	12	No	Yes	No	
Patkar et al. (27)	M	61	Severe depression	Elevated IP	Astrocytoma	Unknown	Left temporal lobe	Yes	Brief	8	No	Yes	No	Patient received dexamethasone prior ECT
Fischer (28)	F	80	Severe depression	No	Gliolastoma multiforme	Unknown	Corpus callosum	No	Ultrabrief	7	Post-ictal confusion, ataxia	No	Yes	
Perry et al. (29)	F	53	Severe depression	No	Arachnoid cyst	4.0 × 4.4 × 1.6	Left-frontoparietal convex.	Yes	Unknown	11	No	Yes	No	
Perry et al. (29)	M	58	psychosis with agitation	No	Arachnoid cyst	1.3 × 4.3 × 1.4	Left posterior fossa	Yes	Unknown	21	No	Yes	No	
Perry et al. (29)	M	43	Severe depression	No	Arachnoid cyst	2.0 × 2.0 × 2.5	Midline posterior fossa	Yes	Unknown	14	No	Yes	No	
Perry et al. (29)	M	19	Mania with psychosis	No	Arachnoid cyst	3.0 × 5.0 × 4.0	middle cranial fossa	Yes	Unknown	1	No	No	Yes	Patient refused further ECT (reason not stated)
Perry et al. (29)	M	42	Catatonia	No	Arachnoid cyst	2.6 × 1.2 × 2.9	Left posterior fossa	No	Unknown	5	No	Yes	No	
Perry et al. (29)	M	44	Severe depression with psychosis	No	Arachnoid cyst	1.3 × 3.5 × 2.3	Middle cranial fossa	Yes	Unknown	5	No	Yes	No	
Huang et al. (30)	F	58	Severe depression	No	Acoustic neuroma	3.3 at CP angle	Left cerebello-pontine angle	No	Brief	6	Post-ictal confusion, delirium, nausea, hearing impairment	No	Yes	
Desseilles et al. (31)	M	58	Severe depression	No	Arachnoid cyst	7.6 × 4.1 × 8.1	Right anterior temporal reg.	Yes	Brief	7	No	Yes	No	
Hanretta et al. (32)	F	29	Severe depression with psychosis	No	Arachnoid cyst	2.5 × 2.5	Right temporal lobe	Yes	Brief	6	No	Yes	No	
Kastenholz et al. (33)	F	21	Catatonia	No	Arachnoid cyst	4.6 × 8.8 × 4.0	Posterior fossa	Yes	Brief	6	No	Yes	No	
Escalona et al. (34)	M	53	Severe depression	No	Arachnoid cyst	Unknown	Left sylvian fissure	Yes	Unknown	Unknown	No	Yes	No	
Restifo and Paterson (35)	M	22	Psychosis	No	Colloid cyst	Unknown	Third ventricle	Yes	Unknown	9	No	Yes	No	
Grover et al. (36)	F	57	Severe depression with psychosis	No	Arachnoid cyst	3.4 × 1.6	Left temporal region	No	Unknown	12	Post-ictal confusion	Yes	No	
Mckinney et al. (37)	F	66	mania	No	Meningioma	1.0	cerebellum	Yes	Brief	8	No	Yes	No	

Outcome data were determined by scoring several questions:

- Were there any neurological symptoms present before ECT?
- Was the presence of the tumor known before ECT?
- What was the type of ECT used—pulse width, electrode placement, dosage, titration strategy?
- Were there any AEs present after ECT, especially neurological and lateralizing symptoms?
- Was ECT discontinued due to the presence of any AEs?
- What was the indication for ECT?
- Was the application of ECT beneficial for the patients according to the authors of the respective case report?
- How many sessions of ECT were applied?
- Were any precautionary measures undertaken if the presence of the brain tumor was known before ECT application?

Despite the large variation in quality and quantity of the information provided, we were also able to sort patients by their biological sex, age, type, localization, and size of the tumor.

## Study Limitations

The main bias of our review is the fact that the topic of ECT and brain tumor is likely underreported in general. The same weakness was pointed out by Maltbie et al. (1) in their original study from 1980. It is very likely that a substantial amount of patients with an undiagnosed brain tumor undergo this treatment without any observable side effects and are therefore not reported in scientific literature. On the other hand, it is possible that AEs after ECT treatment in this subgroup of patients are also underreported, as the newer case reports after 1980 consist largely of patients with clinically insignificant tumors. More advanced investigations, such as the analysis of registry-based data [health maintenance organization (HMO)/insurance claims, government regulation data] could provide a more clear and complex view of this topic.

Another weakness is the varied quality of the presented case reports. In particular, the authors generally did not specify as to how exactly the AEs were monitored after ECT (nor to what extent the patients were neurologically examined prior to ECT). It is therefore possible that these patients did manifest discreet symptoms that were not noticed and only the more obvious AEs were reported.

## Results

Starting from the first case report by Hsiao and Evans (14) in 1984 up to our own in 2019, we reviewed a total of 40 patients, which represents a comparable size-sample to the one analyzed by Maltbie et al. (1) between the years 1945 and 1980 (total of 35). In contrast with the previous review, the presence of a brain tumor was known before ECT in 29 patients (72.5%). Brief pulse width ECT was administered to 28 patients, ultrabrief pulse width to three, and in nine patients, we were unable to trace the pulse width.

The most common indication for ECT in this group of patients was severe depression (18 patients−45%) and psychosis, usually associated with a severe mood disorder (18 patients−45%). Three patients were indicated for ECT due to symptoms of catatonia and one patient for mania.

The most common type of tumor was a meningioma, which was present in 16 patients (40%), with arachnoid cysts coming in second place with 11 patients (27.5%).

Six patients (15% of the sample) manifested AEs after ECT. Out of this group, four patients developed postictal confusion; one patient manifested a set of several unusual, but reversible, symptoms (secondary myoclonic seizure with lateralization unresponsive to intravenous diazepam application, ping-pong gaze, and Todd's paralysis); and one patient was observed to develop a 3-day-long delirium with hearing impairment. All reported AEs were reversible.

ECT was discontinued in six patients (15%)—the first described by Holroyd (22) in 1993, where the treatment was stopped immediately after a random CT scan revealed a tumor in the pituitary area. Ironically, the patient did not show any AEs during ECT administration, and at the time, it was stopped only due to the previous series of negative case reports on the topic. The second cessation of ECT was in the case of an 80-year-old woman due to terminal-stage cancer (24). In another two cases, the ECT was canceled due to lack of effect and refusal to continue with the procedure from the side of the patient (28, 29). Huang et al. (30) described a patient who developed severe AEs after ECT including nausea, hearing loss, and a 3-day delirium—the patient was later diagnosed with acoustic neuroma, and ECT was discontinued. And finally, in our department, we halted ECT in a female patient who manifested a secondary seizure and was subsequently found to have an intracranial tumor in the left parietal lobe (25).

Five patients (12.5%) had neurological symptomatology prior to the administration (including one report of a patient with an elevated intracranial pressure), but none of them manifested any AEs following ECT administration. Five patients were premedicated with dexamethasone to prevent acute edema or phenytoin to prevent secondary seizures.

According to the authors of the respective case reports, ECT was beneficial for 36 patients (90%). ECT was ineffective in three cases and discontinued in one patient after he refused to undergo further treatment (the reason behind this decision was not stated).

Only one patient (27) was reported to have had elevated intracranial pressure prior to the procedure, and he underwent ECT without any AEs.

In this group of patients, we were able to determine the exact number of ECT sessions in 36 patients. The average number of sessions per patient was 9.47.

No patient was reported to have died as a result of ECT application in this sample. The average age of patients in this group was 59.72 years.

## Discussion

There seems to be a clear difference between the two similarly sized groups from 1945 to 1980 and 1984 to 2019. However, our results must also be seen as biased—the topic of ECT and brain tumor is likely in general underreported; in the majority (72.5%) of these new case reports, the presence of the brain tumor was known prior to ECT application which had allowed undertaking specialized precautions (23) (dexamethasone or phenytoin administration) in a substantial number of patients (12.5%), and last but not least, 40% of these reports constitute patients with benign meningiomas or clinically insignificant tumors, which is in contrast to the group of patients reported on by Maltbie et al. (1), where this group represents only seven patients (20%). It should be noted that the latter review consisted of a substantial amount of patients with more aggressive and clinically more significant tumors, such as gliomas (Table 1). We also point out a difference between patients with neurological symptoms manifesting prior to ECT administration-−12.5% of patients in the new group and 45.7% in the Maltbie et al. (1) sample.

Despite this bias, the case reports between 1984 and 2019 demonstrate that ECT can be applied safely in certain patients with an intracranial mass. The average number of ECT sessions was 9.47 in this group compared to 4.57 in the older group. Six patients manifested AEs after the treatment—out of these, only two patients discontinued treatment due to their presence. All AEs in the 1984–2019 sample were reversible, and the majority constituted of patients who were confused after the procedure—an AE not that rare even in patients undergoing ECT without a brain tumor.

We know that seizure activity increases blood pressure and cerebral blood flow, which can lead to an increased edema around the tumor and subsequently to an increase in intracranial pressure and eventually manifestation of neurological signs. This mechanism was first proposed by Carter (38) in 1977 and considered to be the likely cause of the more prominent AEs in this review reported by Huang et al. (30) and Buday et al. (25).

Dexamethasone was used in a substantial amount of patients (12.5%) to reduce the risk of this pathophysiological mechanism in the newer set of case reports. Phenytoin was administered to one patient to prevent a prolonged seizure. None of these individuals manifested AEs; it is therefore possible that the administration of these substrates prior to ECT may reduce the risk of an acute edema and prolonged seizure, respectively.

In their review, Maltbie et al. (1) suggested that more invasive and aggressive tumors might result in a higher incidence of AEs during ECT. The majority of patients in the newer case reports had benign, small, and clinically insignificant tumors, which seem to support the conclusion that this type of lesion poses a minimal risk increase in regard to ECT application. However, as several new case reports mention in this review, some patients with a brain tumor (especially a previously undiagnosed lesion) can manifest reversible, but unusual and concerning, AEs. Currently, neuroimaging is not mandatory before initiating ECT—it was reported several times that the yield of organic pathology if performed in all patients before undergoing ECT is very low (34, 39, 40). We suggest that any new neurological symptoms manifesting during and after ECT (such as a significant lateralization of the seizure, Todd's paralysis, aphasia, eye-movement disorders, secondary seizures, etc.) should prompt a complex neurological investigation and neuroimaging to exclude the presence of an intracranial mass (which might, depending on its localization, play a role in the psychopathology due to which ECT was indicated in the first place), another organic disease of the central nervous system, or even an acute problem, such as intracranial bleeding.

We must also consider that technological advancement and modern titration strategies that seek to individualize the dosage in each patient might also be one of the reasons why there are less and far more benign and reversible reported AEs (41). Unfortunately, in most cases between 1945 and 1980, it was not possible to track the type of devices, titration strategy, and dosage levels used during ECT. Based on the review of case reports emerging since 1984, it seems that the application of ECT is safer than it was originally presumed by Maltbie et al. (1) in 1980, and this method can be applied safely on a case-by-case basis.

## Conclusion

We have reviewed a total of 33 articles of 75 individual patients who underwent ECT in the presence of a brain tumor over the last 80 years. Mounting case reports from 1984 show that this method can be safely administered in patients with benign, small, and otherwise clinically insignificant tumors. The application of ECT in this type of patients should be considered in a case-by-case basis after interdisciplinary consultation with a neurologist and neurosurgeon with a risk/benefit assessment. ECT practitioners should be vigilant of any AEs (especially neurological) manifesting during and after the procedure. The occurrence of such abnormal symptoms should prompt an immediate neurological investigation and proper imaging studies to exclude any underlying organic pathology. Certain precautionary measures, such as dexamethasone or phenytoin application before ECT, could lead to a further minimalization of AEs.

## Author Contributions

All authors listed have made a substantial, direct and intellectual contribution to the work, and approved it for publication.

## Conflict of Interest

The authors declare that the research was conducted in the absence of any commercial or financial relationships that could be construed as a potential conflict of interest.
